# Nicotine oxidation by genetic variants of CYP2B6 and in human brain microsomes

**DOI:** 10.1002/prp2.468

**Published:** 2019-03-11

**Authors:** Adam Joseph Bloom, Pan‐Fen Wang, Evan D. Kharasch

**Affiliations:** ^1^ Department of Psychiatry and Anesthesiology Washington University St. Louis Missouri; ^2^ Department of Anesthesiology Duke University School of Medicine Durham North Carolina

**Keywords:** brain, CYP2B6, microsomes, nicotine

## Abstract

Common variation in the *CYP2B6* gene, encoding the cytochrome P450 2B6 enzyme, is associated with substrate‐specific altered clearance of multiple drugs. CYP2B6 is a minor contributor to hepatic nicotine metabolism, but the enzyme has been proposed as relevant to nicotine‐related behaviors because of reported *CYP2B6* mRNA expression in human brain tissue. Therefore, we hypothesized that CYP2B6 variants would be associated with altered nicotine oxidation, and that nicotine metabolism by CYP2B6 would be detected in human brain microsomes. We generated recombinant enzymes in insect cells corresponding to nine common *CYP2B6* haplotypes and demonstrate genetically determined differences in nicotine oxidation to nicotine iminium ion and nornicotine for both (S) and (R)‐nicotine. Notably, the CYP2B6.6 and CYP2B6.9 variants demonstrated lower intrinsic clearance relative to the reference enzyme, CYP2B6.1. In the presence of human brain microsomes, along with nicotine‐*N*‐oxidation, we also detect nicotine oxidation to nicotine iminium ion. However, unlike *N*‐oxidation, this activity is NADPH independent, does not follow Michaelis‐Menten kinetics, and is not inhibited by NADP or carbon monoxide. Furthermore, metabolism of common CYP2B6 probe substrates, methadone and ketamine, is not detected in the presence of brain microsomes. We conclude that CYP2B6 metabolizes nicotine stereoselectively and common CYP2B6 variants differ in nicotine metabolism activity, but did not find evidence of CYP2B6 activity in human brain.

**Abbreviations**
CL_int_: intrinsic clearance
CYP: cytochrome P450
FMO: flavin‐containing monooxygenase
HBM: human brain microsomes
NADPH: nicotinamide adenine dinucleotide phosphate
POR: P450 oxidoreductase
*V*_max_: maximum velocity

## 1 INTRODUCTION

**Figure 1:**
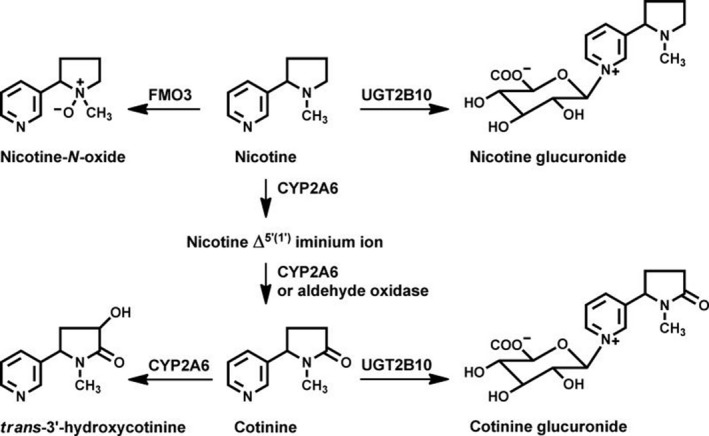
Nicotine metabolism pathways

Tobacco consumption and liability to nicotine dependence are heritable traits.^1^, ^2^ One clearly implicated pathway is the C‐oxidation of nicotine to nicotine iminium ion and subsequently to cotinine by CYP2A6, one of three nicotine metabolism pathways (Figure 1). Variation in nicotine C‐oxidation influences cigarette consumption among dependent smokers and consequent disease risk. CYP2A6 is responsible for 70%‐80% of nicotine metabolism in most people,^3^ and the mechanism of influence is straightforward: smokers with reduced CYP2A6 function metabolize nicotine more slowly, delaying the onset of the withdrawal symptoms that compel nicotine addicts to reach for the next cigarette. Hence, polymorphisms in *CYP2A6* causing slower nicotine metabolism are also associated with reduced cigarette consumption and disease risk in multiple populations.^4^, ^5^, ^6^, ^7^ Nicotine is also metabolized via two other major pathways: glucuronidation by UGT2B10, and oxidation to nicotine‐*N*‐oxide by the flavin‐containing monooxygenases (FMOs). Variation in *FMO3* is associated with altered hepatic nicotine metabolism.^8^

CYP2B6 is a minor contributor to hepatic nicotine metabolism with a *K*_*m*_ approximately 10‐fold higher than that of CYP2A6.^9^ Genetic variation in the *CYP2B6* locus has a small but clinically insignificant influence on in vivo nicotine clearance in most subjects,^10^, ^11^ but may be important in CYP2A6 slow metabolizers. CYP2B6 has also been reported to be expressed in human brain tissue,^12^ unlike CYP2A6, and it has therefore been proposed that genetic variation affecting the CYP2B6 enzyme might play a significant role in nicotine‐related phenotypes.^13^ *CYP2B6* is highly polymorphic, and common haplotypes, especially CYP2B6*6 and *9, containing 516G>T (Q172H, minor allele frequency 26% in European Americans, 37% in African Americans (http://evs.gs.washington.edu/EVS/)) are associated with clinically significantly altered pharmacokinetics for different substrates, relative to the reference allele.^14^, ^15^, ^16^, ^17^, ^18^, ^19^ Because the effects of *CYP2B6* polymorphisms on CYP2B6 activity is substrate‐specific, activities of different CYP2B6 isoforms cannot be assumed for untested substrates, such as nicotine. Therefore, we have expressed polymorphic CYP2B6 enzymes and measured their nicotine metabolism activity in vitro. We further attempted to measure cytochrome P450‐mediated nicotine metabolism in human brain microsomes, where FMO activity was previously demonstrated,^8^, ^20^ using nicotine and other CYP2B6 probe substrates.

## 2 MATERIALS AND METHODS

CYP2B6 variants, reference allele P450 oxidoreductase (POR), and reference allele cytochrome *b*_5_ vectors were previously generated, and recombinant proteins previously expressed in insect cells, as described.^17^, ^21^ Haplotype selection was based on the known clinical significance and frequency of the polymorphism, and a desire to inform the mechanistic basis for the influence of these polymorphisms on metabolism. There is growing evidence that the influence of *CYP2B6* polymorphisms varies between substrates, and may vary between enantiomers of those substrates. For example, 516G>T (Q172H) is a canonical single nucleotide polymorphism (SNP) thought to result in diminished metabolism,^17^, ^21^, ^22^ while 785A>G (K262R) alone causes increased activity for some but not all 2B6 substrates.^21^, ^23^ To evaluate contribution from the two major SNPs, we selected variants with various combinations of these mutations (2B6*4, *6, *7, *9, *16, *19, *26) even though some are minor in population frequency. The other three *2B6* variants (*5, *17, *18) have relatively high allele frequency.

Human brain microsomes were prepared as described^8^ from de‐identified cerebral cortex flash‐frozen postmortem obtained from the National Disease Research Interchange (NDRI), Philadelphia PA, USA. They included four Caucasian males, one Caucasian female, and one African American male with ages ranging from 58 to 90 years. None were reported to be current smokers or substance abusers. In brief, tissues were homogenized with a glass‐teflon homogenizer in ice‐cold potassium phosphate buffer pH 7.4, and centrifuged 20 minute at 9000*g*. The S9 fraction was centrifuged 60 minute at 100 000*g* to obtain a microsomal pellet which was resuspended, further homogenized, centrifuged, and resuspended in buffer containing 20% glycerol^24^, ^25^ for storage at −80°C. Total brain protein concentrations were determined using Bio‐Rad Protein Assay Dye Regent Concentrate which is based on Bradford method. Results were expressed as fmol min^−1^ mg^−1^ of total brain protein, as CYP2B6 protein was not detectable.

### 2.1 Cytochrome P450 and *b*_5_ concentration and POR activity determination

All assays for P450 content, *b*_5_ content, and POR activity were carried out using a Synergy MX Microplate Reader (Biotek, Winooski, VT). Total protein concentrations were determined using Bio‐Rad Protein Assay Dye Regent Concentrate which is based on Bradford method. P450 concentration was determined by difference spectrum of ferrous‐carbon monoxide complex in a CO binding assay (reduced vs reduced CO) using an extinction coefficient De_450‐490 nm_ of 91 mmol L^−1^ cm^−1^. Cytochrome *b*_5_ content was determined by difference spectrum of NADH‐reduced and oxidized *b*_5_ using an extinction coefficient De_424‐410 nm_ of 185 m mol L^−1^ cm^−1^.

P450 oxidoreductase activity was measured by NADPH‐cytochrome c reductase activity. To 200 μL of diluted cell lysate in 0.3 mol L^−1^ potassium phosphate buffer at pH 7.7 containing 40 μmol L^−1^ cytochrome c, 100 μmol L^−1^ NADPH was added to initiate the reduction of cytochrome c and the reaction was followed at 550 nm and 23°C. The reaction rate was calculated using an extinction coefficient of e_550 nm_ of 21 mmol L^−1^ cm^−1^ for reduced cytochrome c. POR activity was converted to POR content based on the assumption that 3000 nmol of cytochrome c is reduced/min per nmol POR at 23°C.^17^, ^26^

### 2.2 Substrate metabolism

All incubations were carried out in 96‐well PCR plates in 50 μL total volume at 37°C, as previously described.^8^ Incubations with recombinant CYP2B6/POR/b5 proceeded for 10 minutes and incubations with human brain microsomes proceeded for 1 hour, unless otherwise noted, following a 2‐minute preincubation and initiation with 25, 50, 100, 250, 500, 1000, 1,500, or 2,000 μmol L^−1^ S‐nicotine or R‐nicotine (Toronto Research Chemicals, Toronto, Canada) in the presence of 1 mmol L^−1^ NADPH (Sigma‐Aldrich, St. Louis, MO). Inhibition of metabolite formation was tested at 100 μmol L^−1^ nicotine for convenience to ensure metabolite concentrations remained within the concentration curve after incubation of up to 6 hours. Reactions were quenched by adding 10 μL 15% zinc sulfate containing 200 ng mL^−1^ of the internal standard, (d_3_)‐nicotine‐N‐oxide (Toronto Research Chemicals, Toronto, Canada). Samples were centrifuged and supernatants were removed for LC/MS analysis.

Methadone incubations were performed with 10 μmol L^−1^ methadone and processed and analyzed as previously described.^21^, ^27^ In brief, methadone was from the National Institute on Drug Abuse (Bethesda, MD); all other reagents were purchased from Sigma‐Aldrich. Reactions were quenched with 40 μl 20% trichloroacetic acid containing internal standard (d3‐EDDP), and centrifuged for 5 minutes at 2500*g*; the supernatant was processed by solid phase extraction as previously described,^28^, ^29^ except that for Strata‐X‐C 33‐μm, 30 mg/well plates (Phenomenex, Torrance, CA) were used. Analysis was performed on a Sunfire C18 column (2.1 × 50 mm, 3.5‐μm) (Waters, Milford, MA) with a 2‐μm column filter guard (Supleco Analytical, Bellefonte, PA). Sample injections were 25 μL and the column oven was held at 30°C. Mobile phase A was 4.5 mmol L^−1^ ammonium acetate in Milli‐Q water, pH 4.5, and mobile phase B was 4.5 mol L^−1^ ammonium acetate in acetonitrile. The mobile phase gradient (0.4 mL min^−1^) was 25% B for 0.5 minutes, linear gradient to 75% B between 0.5 and 4.2 minutes, held at 75% B until 5.0 minutes, immediately decreased back to 25% B and re‐equilibrated at initial conditions for 3.0 minutes.

Ketamine incubations were performed with 80 μmol L^−1^ ketamine, and processed and analyzed as previously described^17^ In brief, the reactions were quenched with 20% trichloroacetic acid containing norketamine‐d4. The plate was centrifuged at 900 *g* for 5 minutes and the supernatant was subjected to solid phase extraction.^30^ Analytes were eluted with methanol and evaporated to dryness under nitrogen. For analysis, samples were resuspended in mobile phase (10 mmol L^−1^ ammonium acetate, pH 7.6). High performance liquid chromatography (HPLC)‐mass spectrometry analysis was performed on an ultra‐fast liquid chromatography system (Shimadzu Scientific Instruments, Columbia, MD) with a CMB‐20A system controller, two LC‐20ADXR pumps, DGU‐20A3 degasser, SIL‐20AC autosampler, FCV‐11AL solvent selection module, and CTO‐20A column oven (30°C) containing a ChiralPak AGP analytical column (100 × 2.0 mm, 5 μm) and AGP guard column (10 × 2.0 mm) (Chiral Technologies, West Chester, PA), coupled to an API 4000 QTrap LC‐MS/MS linear ion trap triple quadrupole tandem mass spectrometer (Applied Biosystems/MDS Sciex, Foster City, CA). The mobile phase (0.22 mL min^−1^) was 10 mmol L^−1^ ammonium acetate in water, pH 7.60 (A) and isopropanol (B). The column was equilibrated with 4% B, maintained after injection for 0.1 minute, then a linear gradient to 16.8% B applied over 12 minutes, and then reverted back to 4% B over 0.5 minute and re‐equilibrated for 3.5 minutes.

### 2.3 Liquid chromatography tandem mass spectrometry of nicotine metabolites

Nicotine metabolites were analyzed by liquid chromatography tandem mass spectrometry (LC‐MS\MS) based on previous methods^8^ and quantified using the ratio of metabolite to internal standard, subtracting background from control incubations (without NADPH), and using calibration curves. The subtraction of control (NADPH‐) incubations was necessary to subtract trace nicotine metabolites present in the substrate, nicotine. Three replicates were performed for each incubation. Further control incubations for brain microsomes included with boiled protein or without protein.

Liquid chromatography tandem mass spectrometry analyses were performed on an API 4000 Q‐TRAP triple quadrupole mass spectrometer (Applied Biosystems Sciex, Foster City, CA) equipped with an electrospray source. The HPLC system consisted of two LC 20AC pumps with a CTO‐20A column oven, SIL‐20A autosampler, DGU‐20A3 degasser, FCF‐11AL valve, and a CBM 20A controller (Shimadzu, Columbia, MD). The chromatographic separation was performed on an xBridge column (150 × 2.1, 3.5 μm, Waters, Milford, MA) with an inline precolumn 0.2 μm filter. The injection volume was 5 μL and the oven temperature was 40°C. Mobile phase (0.3 mL min^−1^) was (A) 4.5 mmol L^−1^ ammonium acetate pH 4.0 and (B) 4.5 mmol L^−1^ ammonium acetate in acetonitrile using the following program: 1% B for 4 minutes, linear gradient to 25% B between 4 and 5 minutes, held at 25% B until 6.0 minutes, and then re‐equilibrated to initial conditions between 6.5 and 8.5 minutes. Under these conditions, the retention times for nicotine iminium and nornicotine (a minor CYP2B6 nicotine metabolite^9^) were 2.5 and 3.7 minutes, respectively; for *cis* and *trans* nicotine‐*N*‐oxide, they were 4.7 and 5.2 minutes, respectively. The instrument was operated in positive‐ion mode at 450°C with an ion spray voltage of 5500 V, entrance potential of 10 V, and exit potential of 22 V. The curtain gas was set at 20, ion source gas 1 at 30, and ion source gas 2 at 40. Transitions monitored for nicotine iminium were *m*/*z* 161 → 130, for nornicotine, *m*/*z* 149 → 117, for nicotine‐*N*‐oxide *m*/*z* 179 → 132, and for deuterated (d3)‐nicotine‐*N*‐oxide, *m*/*z* 182 → 132.

### 2.4 Statistical analysis

Michaelis‐Menten parameters for recombinant enzyme experiments were determined by nonlinear regression using SigmaPlot (Systat Software, San Jose, CA).

## 3 RESULTS

### 3.1 Nicotine oxidation by variant recombinant CYP2B6 enzymes

**Figure 2:**
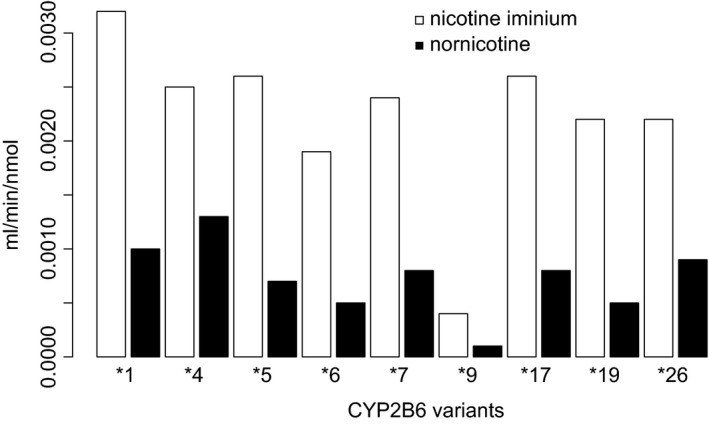
Intrinsic clearance (CL_int_) of (S)‐nicotine to nicotine iminium ion and nornicotine by variant recombinant CYP2B6 enzymes

Nine recombinant versions of the CYP2B6 enzymes, representing common *CYP2B6* haplotypes, were expressed to assess their relative nicotine metabolism activities. Two nicotine metabolites were measured, nicotine iminium ion and nornicotine, with *K*_*m*_ and *V*_max_ parameters determined for both metabolites for each variant (Table 1). Our results confirm the role of CYP2B6 in nornicotine formation.^9^ As expected, the activity of the most common variant enzyme, CYP2B6.6, was lower than the reference allele enzyme, CYP2B6.1, regarding both nicotine iminum (Cl_int_ = 1.9 vs 3.2 μL min^−1^ nmol^−1^) and nornicotine (Cl_int_ = 0.5 vs 1.0 μL min^−1^ nmol^−1^) (Figure 2).

**Table 1:** Kinetic parameters for (S)‐nicotine‐iminium and (S)‐nornicotine formation by variant CYP2B6 proteins

Allele	Protein variant	(S)‐iminium			(S)‐nornicotine			CL_int_ (nornicotine/iminium)
		*K*_*m*_ (μmol L^−1^)	*V*_max_ (pmol min^−1^ pmol^−1^)	CL_int_ (mL^−1^ min^−1^ nmol^−1^)	*K*_*m*_ (μmol L^−1^)	*V*_max_ (pmol min^−1^ pmol^−1^)	CL_int_ (mL min^−1^ nmol^−1^)	
*1		269 ± 37	0.85 ± 0.04	0.0032	184 ± 27	0.19 ± 0.01	0.0010	0.33
*4	K262R	333 ± 32	0.83 ± 0.03	0.0025	167 ± 28	0.21 ± 0.01	0.0013	0.50
*5	R487C	257 ± 22	0.66 ± 0.01	0.0026	132 ± 18	0.09 ± 0.00	0.0007	0.27
*6	Q172H/K262R	307 ± 16	0.58 ± 0.01	0.0019	110 ± 15	0.06 ± 0.00	0.0005	0.29
*7	Q172H/K262R/R487C	236 ± 21	0.56 ± 0.01	0.0024	108 ± 23	0.09 ± 0.00	0.0008	0.35
*9	Q172H	406 ± 44	0.18 ± 0.01	0.0004	134 ± 21	0.02 ± 0.00	0.0001	0.34
*17	T26S/D28G/R29T	332 ± 22	0.85 ± 0.02	0.0026	130 ± 17	0.11 ± 0.00	0.0008	0.33
*19	Q172H/K262R/R336C	271 ± 27	0.60 ± 0.02	0.0022	114 ± 26	0.06 ± 0.00	0.0005	0.24
*26	P167A/Q172H/K262R	189 ± 21	0.41 ± 0.01	0.0022	86 ± 20	0.08 ± 0.00	0.0009	0.43

Variation in CYP2B6 affects enzyme activity differently depending on substrate, which may include differences between isomers of the same molecule. Nicotine occurs in two isomers, though approximately 99% of naturally occurring nicotine derived from tobacco and other plants is the (S) isomer. In order to determine if effects on CYP2B6 nicotine metabolism activity were also enantiomer‐specific, incubations with four CYP2B6 haplotypes were repeated with (R)‐nicotine (Table 2). *K*_*m*_ and *V*_max_ parameters were different for (R)‐nicotine and (S)‐nicotine, but differences between CYP2B6 variants appear consistent, that is, the activity of CYP2B6.6, was again lower than the reference allele enzyme for nicotine iminum (Cl_int_ = 0.8 vs 1.8 μL^−1^ min^−1^ nmol^−1^) and nornicotine (Cl_int_ = 0.1 vs 0.2 μL^−1^ min^−1^ nmol^−1^). Interestingly, the regioselectivity of the enzyme, that is, the metabolite ratio of nicotine iminium ion vs nornicotine, was relatively consistent among different CYP2B6 variants, but differed between (S)‐ and (R)‐nicotine (Tables 1 and 2).

**Table 2:** Kinetic parameters for (R)‐nicotine‐iminium and (R)‐nornicotine formation by variant CYP2B6 proteins

Allele	Protein variant	(R)‐iminium			(R)‐nornicotine			CL_int_ (nornicotine/iminium)
		*K*_*m*_ (μmol L^−1^)	*V*_max_ (pmol min^−1^ pmol^−1^)	CL_int_ (mL min^−1^ nmol^−1^)	*K*_*m*_ (μmol L^−1^)	*V*_max_ (pmol min^−1^ pmol^−1^)	CL_int_ (mL min^−1^ nmol^−1^)	
*1		1537 ± 352	2.78 ± 0.24	0.0018	1288 ± 289	0.25 ± 0.03	0.0002	0.11
*4	K262R	1602 ± 436	2.11 ± 0.32	0.0013	936 ± 314	0.15 ± 0.02	0.0002	0.11
*6	Q172H/K262R	918 ± 409	0.73 ± 0.15	0.0008	956 ± 584	0.07 ± 0.02	0.0001	0.10
*9	Q172H	5040 ± 2276	0.72 ± 0.25	0.0001	1435 ± 665	0.03 ± 0.01	0.0000	0.13

### 3.2 CYP2B6 substrate metabolism by human brain microsomes

**Figure 3:**
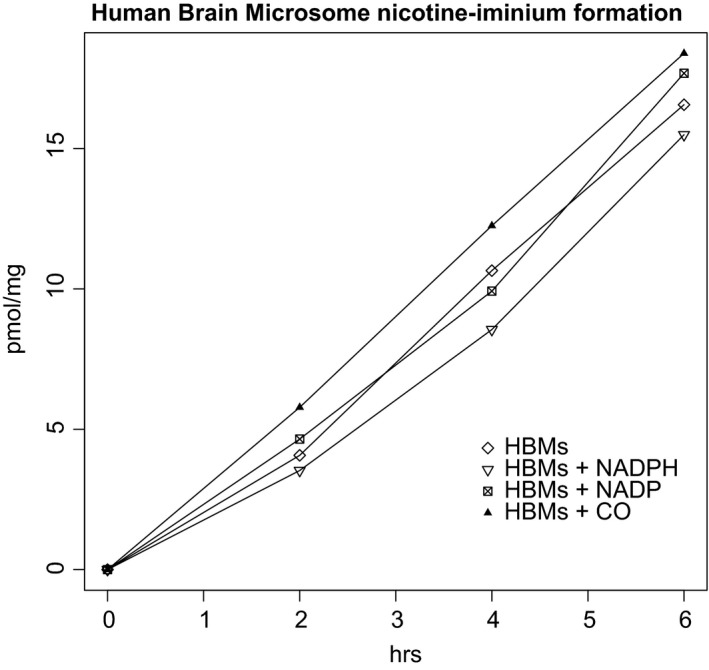
Nicotine iminum formation over time in the presence of human brain microsomes (HBMs), with and without NADPH, inhibited by NADP or following carbon monoxide (CO) treatment

**Figure 4:**
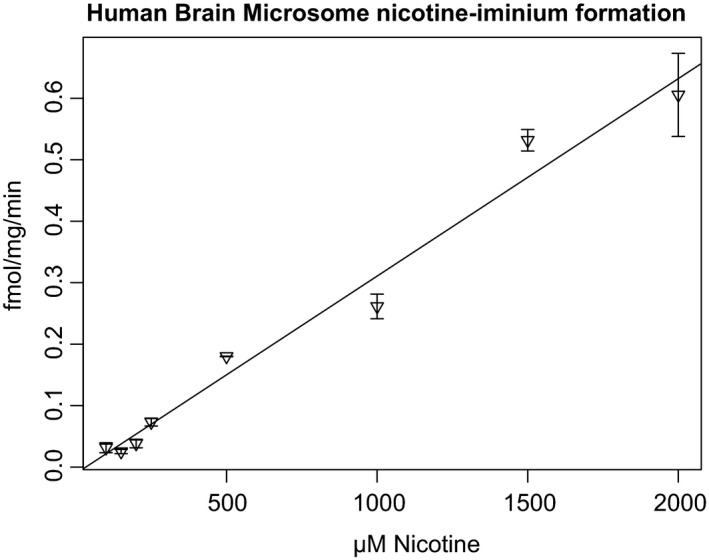
Nicotine iminum ion formed per mg human brain microsomal protein per minute

Microsomes were prepared from the cerebral cortices of six postmortem individuals. Nicotine‐*N*‐oxidation by FMO enzymes was used as a positive control for tissue quality because FMO enzyme activity is diminished by heat treatment at room temperature or repeated freeze‐thawing. Nicotine‐iminium, but not nornicotine, was detected in incubations with all six samples. In the presence of 100 μmol L^−1^ nicotine, the average turnover was 264 ± 133 (range 142‐498) fmol min^−1^ mg^−1^ of microsomal protein. Activity was not significantly correlated with age (mean 72.9 ± 11.6 years) or postmortem interval (mean 11.6 ± 3.1 hours). The activity remained linear for at least 6 hours (Figure 3). Incubation conditions were interrogated to determine whether iminium production was consistent with cytochrome P450 activity. The activity was abolished by heat‐treatment (65°C 1 hour), but did not require NADPH, and was not inhibited by NADP (2 mmol L^−1^) or carbon monoxide treatment. Nicotine‐*N*‐oxide, the product of FMO enzymes (Figure 1), was also detected in the microsome incubations, but its formation required NADPH. On further characterizing one brain, we found the NADPH‐independent nicotine‐iminium formation did not follow Michaelis‐Menten kinetics (Figure 4). Cotinine and hydroxycotinine were also not generated in any human brain incubation.

To further investigate possible CYP2B6 activity in human brain microsomes, we incubated them with two additional common CYP2B6 probe substrates, methadone (10 μmol L^−1^) and ketamine (80 μmol L^−1^), the approximate *K*_*m*_ for CYP2B6 for each substrate.^27^, ^31^ Neither 2‐ethylidene‐1,5‐dimethyl‐3,3‐diphenylpyrrolidine (EDDP) nor norketamine were detected, respectively.

## 4 DISCUSSION

Extensive evidence demonstrates that functional variation in cytochrome P450 genes can significantly affect the metabolism of many small molecules including pharmaceuticals and toxins. Importantly, common functional polymorphisms in *CYP2B6* alter enzyme activity in a substrate‐dependent manner,^15^, ^16^, ^19^, ^22^, ^23^, ^32^, ^33^, ^34^ and thus it is important to evaluate these enzyme variants for any CYP2B6 substrate. Here, we demonstrate how these common variants also affect nicotine metabolism in vitro. The differences we report, both for (S)‐nicotine and (R)‐nicotine, confirm the expectation of altered activities for common variants including CYP2B6.6 and CYP2B6.9, relative to the reference allele enzyme, CYP2B6.1. Because (S)‐nicotine is the common naturally occurring form of nicotine relevant to smoking behavior, experiments with (R)‐nicotine were only performed to explore substrate specificity and stereoselectivity. The activities of the nine enzyme variants also differed with regard to the relative metabolite ratios of (S)‐nicotine iminium and (S)‐nornicotine. The differences in this ratio demonstrate altered regioselectivity associated with each polymorphism, that is, changes in the activity of each enzyme isoform depend on which region of the substrate molecule the enzyme oxidizes,^35^ resulting in the formation of nicotine iminum vs nornicotine.

Of particular interest are the differences in activity related to the two amino acid changes, Q172H and K262R, which individually define the CYP2B6*9 and CYP2B6*4 variants respectively, and together define the very common CYP2B6*6 haplotype. For most substrates, activity of the CYP2B6.9 variant is exceptionally low, activity of the CYP2B6.4 variant is similar or greater than that of the reference allele, while the activity of CYP2B6.6 lies between CYP2B6.4 and CYP2B6.9, depending on substrate.^14^, ^21^, ^22^ This demonstrates that the K262R amino acid change can partially compensate for the Q172H loss‐of‐function, depending on substrate, and we find this to be the case for nicotine as well. CYP2B6*6 is the most commonly studied variant haplotype because of its high population frequency and has been associated with low activity in vivo.^16^, ^33^ However, it is also important to note that part of this loss of activity may be due to differences in mRNA or protein expression or splicing, and not strictly related to catalytic activity.^10^, ^36^, ^37^ CYP2B6*6 (and CYP2B6*9) have also been reportedly associated with more rapid in vivo nicotine metabolism.^34^

We previously demonstrated that common *CYP2B6* haplotypes, including missense variants and noncoding variants that affect gene splicing, are associated with differences in in vivo nicotine metabolism.^10^ However, the small contribution of CYP2B6 to hepatic nicotine metabolism^38^ is unlikely to influence smoking behaviors. *CYP2B6* mRNA expression has been reported in human brain,^12^ although direct evidence of CYP2B6 activity in brain tissue was not previously demonstrated. It has therefore been suggested that genetically determined variation in CYP2B6 activity in extrahepatic tissues might influence smoking behavior by altering local nicotine levels in the brain.^13^ We recently demonstrated nicotine‐*N*‐oxidation by flavin‐containing monoxygenases in microsomes prepared from human brain.^8^ This provided an opportunity to further investigate possible cytochrome P450‐related nicotine metabolism in postmortem samples known to retain detectable microsomal enzyme activity. Given the difficulty of obtaining postmortem human brain material, it was important to have a positive control for tissue quality. Postmortem intervals before initial freezing were typically 12 hours or more, and FMO activity is also subject to degradation at room temperature and following repeated freeze‐thawing.

Interestingly, we found nicotine iminium ion produced in the presence of human brain microsomes (nornicotine was not detected), but the mechanism of this weak activity was obscure. It is independent of the cofactor NADPH and not inhibited by either carbon monoxide or NADP, though the activity was abolished by heat treatment of the microsomes. Meanwhile, the production of nicotine‐*N*‐oxide in the same incubations required NADPH and was inhibited by carbon monoxide, NADP, and heat treatment, as expected. We therefore conclude that the iminium ion detected was not due to cytochrome P450s or any typical enzymatic activity. NADP and carbon monoxide are general inhibitors of CYP enzymes. Had either general inhibitor abolished the activity, we would have pursued other specific inhibitors to narrow down the source of the activity to specific enzymes. Given the high *K*_*m*_ of CYP2B6 for nicotine, we further probed human brain microsomes with two common CYP2B6 substrates, ketamine and methadone, and found no evidence of metabolism of either.

Variation in local nicotine metabolism in the brain is ultimately interesting because of the potential for targeting these pathways to aid smoking cessation. Our prior work suggested that differences in nicotine metabolism within the brain might influence nicotine dependence in ways qualitatively different from the influence of hepatic metabolism; namely, common functional variation in an enzyme active in both liver and brain, FMO3, is associated with a significant difference in nicotine dependence *independent* of cigarette consumption level. Meanwhile, variation in the primary cytochrome‐P450‐mediated nicotine metabolism pathway affects consumption among dependent smokers but not liability to become dependent.^8^, ^39^ It was therefore important to understand whether the divergent influences of these two pathways are related to inherent differences in their activities—that is, the metabolites produced—or differences in their tissue distributions. If the highly polymorphic CYP enzymes responsible for nicotine C‐oxidation were active in brain, it would argue against FMO3's local brain activity being the mechanism of its unique influence on dependence. Unlike *CYP2A6, CYP2B6* mRNA expression has been reported in human brain tissue, but aside from its role in bupropion metabolism, genetic variation in *CYP2B6* has not been associated with smoking phenotypes. However, given the well‐documented substrate specificity of functional variation in CYP2B6, it was also necessary to determine which variants affect nicotine metabolism, a question not previously addressed in vitro. A key limitation of our study was the problem of enzymatic stability in postmortem brain tissue. Nevertheless, if undetected CYP2B6 activity does occur in the human brain, with the potential to influence smoking behavior in a similar manner to FMO3, associations between *CYP2B6* genotype and nicotine dependence should be detectable in human genetic studies based on our in vitro activity results together with previously published findings regarding variation in CYP2B6 expression.

In summary, we conclude that nicotine metabolism by CYP2B6 is stereoselective, and common polymorphisms in *CYP2B6* significantly influence the oxidation of both (S) and (R)‐nicotine to nicotine iminium and nornicotine. However, the small contribution of CYP2B6 to nicotine metabolism in the liver, and its undetectable activity in human brain tissue, make it highly unlikely that this genetic variation influences complex smoking behaviors.

## AUTHOR CONTRIBUTIONS

*Participated in research design*: Bloom, Wang, and Kharasch.

*Conducted experiments*: Bloom.

*Contributed new reagents or analytic tools*: Bloom, Wang, and Kharasch.

*Performed data analysis*: Bloom.

*Wrote or contributed to the writing of the manuscript*: Bloom and Kharasch.

## DISCLOSURE

None declared.

## References

[1] Koopmans JR , Slutske WS , Heath AC , Neale MC , Boomsma DI . The genetics of smoking initiation and quantity smoked in Dutch adolescent and young adult twins. Behav Genet. 1999;383‐393. 10857244 10.1023/a:1021618719735

[2] Li MD . The genetics of smoking related behavior: a brief review. Am J Med Sci. 2003;168‐173. 10.1097/00000441-200310000-00003 14557728

[3] Hukkanen J , Jacob P 3rd , Benowitz NL . Metabolism and disposition kinetics of nicotine. Pharmacol Rev. 2005;79‐115. 15734728 10.1124/pr.57.1.3

[4] Cho MH , Castaldi PJ , Wan ES , et al. A genome‐wide association study of COPD identifies a susceptibility locus on chromosome 19q13. Hum Mol Genet. 2012;947‐957. 22080838 10.1093/hmg/ddr524 PMC3298111

[5] Liu T , Xie CB , Ma WJ , Chen WQ . Association between CYP2A6 genetic polymorphisms and lung cancer: a meta‐analysis of case‐control studies. Environ Mol Mutagen. 2013;133‐140. 23203414 10.1002/em.21751

[6] Thorgeirsson TE , Gudbjartsson DF , Surakka I , et al. Sequence variants at CHRNB3‐CHRNA6 and CYP2A6 affect smoking behavior. Nat Genet. 2010;448‐453. 20418888 10.1038/ng.573 PMC3080600

[7] Wassenaar CA , Ye Y , Cai Q , et al. CYP2A6 reduced activity gene variants confer reduction in lung cancer risk in African American smokers–findings from two independent populations. Carcinogenesis. 2015;99‐103. 25416559 10.1093/carcin/bgu235 PMC4291053

[8] Teitelbaum AM , Murphy SE , Akk G , et al. Nicotine dependence is associated with functional variation in FMO3, an enzyme that metabolizes nicotine in the brain. Pharmacogenomics J. 2018;136‐143. 28290528 10.1038/tpj.2016.92 PMC5599305

[9] Yamanaka H , Nakajima M , Fukami T , et al. CYP2A6 AND CYP2B6 are involved in nornicotine formation from nicotine in humans: interindividual differences in these contributions. Drug Metab Dispos. 2005;1811‐1818. 16135656 10.1124/dmd.105.006254

[10] Bloom AJ , Martinez M , Chen LS , Bierut LJ , Murphy SE , Goate A . CYP2B6 non‐coding variation associated with smoking cessation is also associated with differences in allelic expression, splicing, and nicotine metabolism independent of common amino‐acid changes. PLoS ONE. 2013;e79700. 24260284 10.1371/journal.pone.0079700 PMC3829832

[11] Lee AM , Jepson C , Shields PG , Benowitz N , Lerman C , Tyndale RF . CYP2B6 genotype does not alter nicotine metabolism, plasma levels, or abstinence with nicotine replacement therapy. Cancer Epidemiol Biomarkers Prev. 2007;1312‐1314. 17548706 10.1158/1055-9965.EPI-07-0188

[12] Gervot L , Rochat B , Gautier JC , et al. Human CYP2B6: expression, inducibility and catalytic activities. Pharmacogenetics. 1999;295‐306. 10471061

[13] Miksys S , Lerman C , Shields PG , Mash DC , Tyndale RF . Smoking, alcoholism and genetic polymorphisms alter CYP2B6 levels in human brain. Neuropharmacology. 2003;122‐132. 12814665 10.1016/s0028-3908(03)00136-9

[14] Calinski DM , Zhang H , Ludeman S , Dolan ME , Hollenberg PF . Hydroxylation and N‐dechloroethylation of Ifosfamide and deuterated Ifosfamide by the human cytochrome p450s and their commonly occurring polymorphisms. Drug Metab Dispos. 2015;1084‐1090. 25934575 10.1124/dmd.115.063628 PMC4468438

[15] Crane AL , Klein K , Zanger UM , Olson JR . Effect of CYP2B6*6 and CYP2C19*2 genotype on chlorpyrifos metabolism. Toxicology. 2012;115‐122. 10.1016/j.tox.2012.01.006 PMC3935332 22281205

[16] Kharasch ED , Regina KJ , Blood J , Friedel C . Methadone pharmacogenetics: CYP2B6 polymorphisms determine plasma concentrations, clearance, and metabolism. Anesthesiology. 2015;1142‐1153. 10.1097/ALN.0000000000000867 PMC4667947 26389554

[17] Wang PF , Neiner A , Kharasch ED . Stereoselective ketamine metabolism by genetic variants of cytochrome P450 CYP2B6 and cytochrome P450 oxidoreductase. Anesthesiology. 2018;756‐768. 30085944 10.1097/ALN.0000000000002371

[18] Yimer G , Amogne W , Habtewold A , et al. High plasma efavirenz level and CYP2B6*6 are associated with efavirenz‐based HAART‐induced liver injury in the treatment of naive HIV patients from Ethiopia: a prospective cohort study. Pharmacogenomics J. 2012;499‐506. 21862974 10.1038/tpj.2011.34

[19] Yuce‐Artun N , Baskak B , Ozel‐Kizil ET , et al. Influence of CYP2B6 and CYP2C19 polymorphisms on sertraline metabolism in major depression patients. International journal of clinical pharmacy. 2016;388‐394. 26830411 10.1007/s11096-016-0259-8

[20] Bhagwat SV , Bhamre S , Boyd MR , Ravindranath V . Cerebral metabolism of imipramine and a purified flavin‐containing monooxygenase from human brain. Neuropsychopharmacology. 1996;133‐142. 8840349 10.1016/0893-133X(95)00175-D

[21] Gadel S , Friedel C , Kharasch ED . Differences in methadone metabolism by CYP2B6 variants. Drug Metab Dispos. 2015;994‐1001. 25897175 10.1124/dmd.115.064352 PMC4468442

[22] Zhang H , Sridar C , Kenaan C , Amunugama H , Ballou DP , Hollenberg PF . Polymorphic variants of cytochrome P450 2B6 (CYP2B6.4‐CYP2B6.9) exhibit altered rates of metabolism for bupropion and efavirenz: a charge‐reversal mutation in the K139E variant (CYP2B6.8) impairs formation of a functional cytochrome p450‐reductase complex. J Pharmacol Exp Ther. 2011;803‐809. 21659470 10.1124/jpet.111.183111 PMC3164347

[23] Honda M , Muroi Y , Tamaki Y , et al. Functional characterization of CYP2B6 allelic variants in demethylation of antimalarial artemether. Drug Metab Dispos. 2011;1860‐1865. 21746968 10.1124/dmd.111.040352

[24] Raccor BS , Claessens AJ , Dinh JC , et al. Potential contribution of cytochrome P450 2B6 to hepatic 4‐hydroxycyclophosphamide formation in vitro and in vivo. Drug Metab Dispos. 2012;54‐63. 21976622 10.1124/dmd.111.039347 PMC3250049

[25] Volak LP , Ghirmai S , Cashman JR , Court MH . Curcuminoids inhibit multiple human cytochromes P450, UDP‐glucuronosyltransferase, and sulfotransferase enzymes, whereas piperine is a relatively selective CYP3A4 inhibitor. Drug Metab Dispos. 2008;1594‐1605. 18480186 10.1124/dmd.108.020552 PMC2574793

[26] Guengerich FP , Martin MV , Sohl CD , Cheng Q . Measurement of cytochrome P450 and NADPH‐cytochrome P450 reductase. Nat Protoc. 2009;1245‐1251. 19661994 10.1038/nprot.2009.121 PMC3843963

[27] Gadel S , Crafford A , Regina K , Kharasch ED . Methadone N‐demethylation by the common CYP2B6 allelic variant CYP2B6.6. Drug Metab Dispos. 2013;709‐713. 23298862 10.1124/dmd.112.050625 PMC3608451

[28] Kharasch ED , Hoffer C , Whittington D , Sheffels P . Role of hepatic and intestinal cytochrome P450 3A and 2B6 in the metabolism, disposition, and miotic effects of methadone. Clin Pharmacol Ther. 2004;250‐269. 15371986 10.1016/j.clpt.2004.05.003

[29] Kharasch ED , Neiner A , Kraus K , et al. Bioequivalance and therapeutic equivalence of generic and brand bupropion in adults with major depression: a randomized clinical trial. Clin Pharmacol Ther. 2018; [Epub ahead of print]. 10.1002/cpt.1309 PMC6465131 30460996

[30] Rao LK , Flaker AM , Friedel CC , Kharasch ED . Role of cytochrome P4502B6 polymorphisms in ketamine metabolism and clearance. Anesthesiology. 2016;1103‐1112. 27763887 10.1097/ALN.0000000000001392

[31] Li Y , Coller JK , Hutchinson MR , et al. The CYP2B6*6 allele significantly alters the N‐demethylation of ketamine enantiomers in vitro. Drug Metab Dispos. 2013;1264‐1272. 23550066 10.1124/dmd.113.051631

[32] Desta Z , Saussele T , Ward B , et al. Impact of CYP2B6 polymorphism on hepatic efavirenz metabolism in vitro. Pharmacogenomics. 2007;547‐558. 17559344 10.2217/14622416.8.6.547

[33] Lv J , Hu L , Zhuo W , Zhang C , Zhou H , Fan L . Effects of the selected cytochrome P450 oxidoreductase genetic polymorphisms on cytochrome P450 2B6 activity as measured by bupropion hydroxylation. Pharmacogenet Genomics. 2016;80‐87. 26580670 10.1097/FPC.0000000000000190

[34] Ring HZ , Valdes AM , Nishita DM , et al. Gene‐gene interactions between CYP2B6 and CYP2A6 in nicotine metabolism. Pharmacogenet Genomics. 2007;1007‐1015. 18004205 10.1097/01.fpc.0000220560.59972.33

[35] Olah J , Mulholland AJ , Harvey JN . Understanding the determinants of selectivity in drug metabolism through modeling of dextromethorphan oxidation by cytochrome P450. Proc Natl Acad Sci USA. 2011;6050‐6055. 21444768 10.1073/pnas.1010194108 PMC3076858

[36] Burgess KS , Ipe J , Swart M , et al. Variants in the CYP2B6 3'UTR alter in vitro and in vivo CYP2B6 activity: potential role of MicroRNAs. Clin Pharmacol Ther. 2018;130‐138. 28960269 10.1002/cpt.892 PMC5871545

[37] Hofmann MH , Blievernicht JK , Klein K , et al. Aberrant splicing caused by single nucleotide polymorphism c.516G>T [Q172H], a marker of CYP2B6*6, is responsible for decreased expression and activity of CYP2B6 in liver. J Pharmacol Exp Ther. 2008;284‐292. 10.1124/jpet.107.133306 18171905

[38] Al Koudsi N , Tyndale RF . Hepatic CYP2B6 is altered by genetic, physiologic, and environmental factors but plays little role in nicotine metabolism. Xenobiotica. 2010;381‐392. 20307138 10.3109/00498251003713958

[39] Bloom AJ , Harari O , Martinez M , et al. Use of a predictive model derived from in vivo endophenotype measurements to demonstrate associations with a complex locus, CYP2A6. Hum Mol Genet. 2012;3050‐3062. 22451501 10.1093/hmg/dds114 PMC3373237

