# Supplementary figures and images for: Nicotine oxidation by genetic variants of CYP2B6 and in human brain microsomes
Source: Pharmacol Res Perspect. 2019 Mar 11;7(2):e00468. doi: 10.1002/prp2.468 (PMC6411694; doi:10.1002/prp2.468)

# Human Brain Microsome cis+trans NNO formation

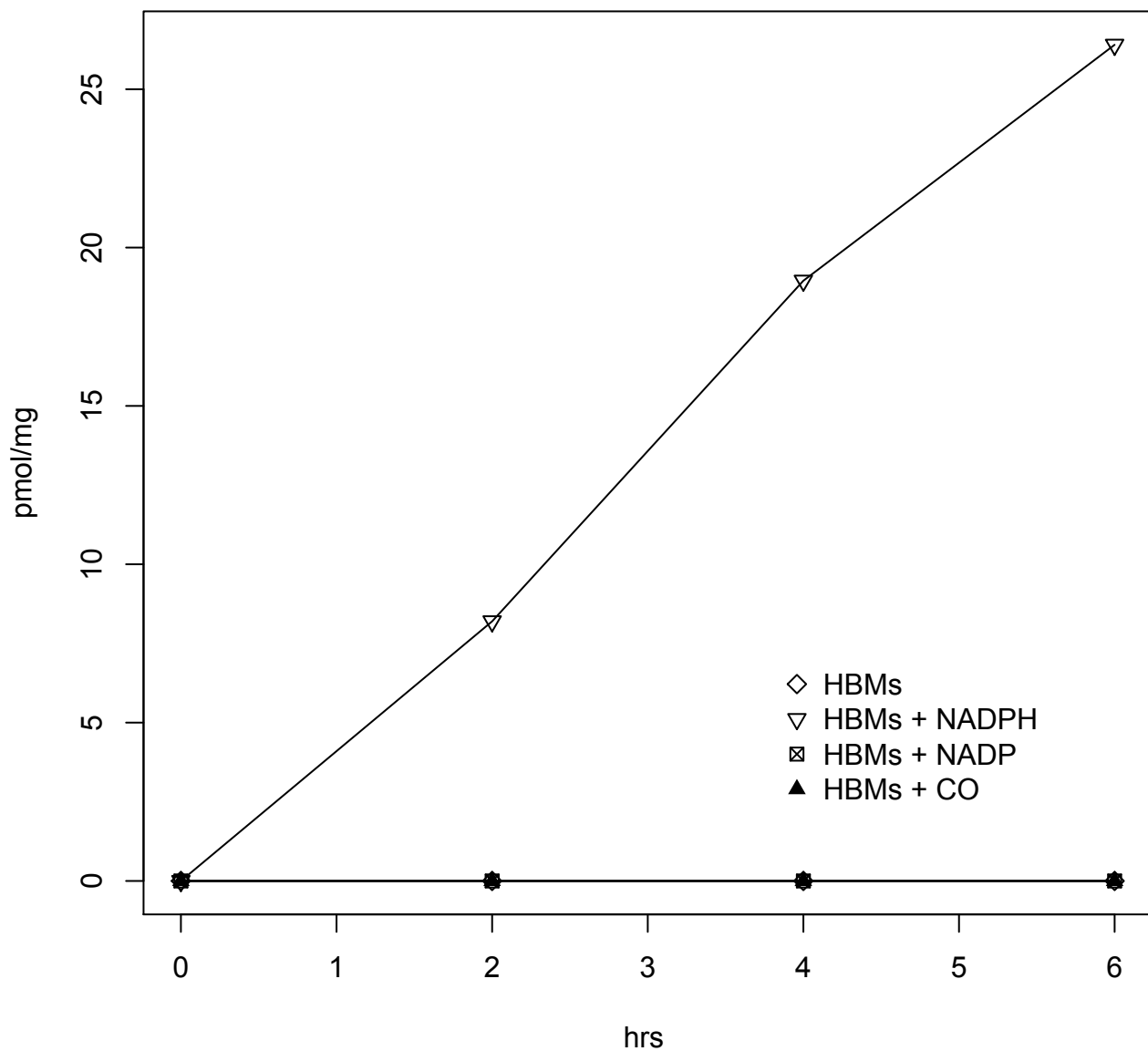

Supplement: Supplementary file 1 [file PRP2-7-e00468-s001.pdf]
